# Cerebral perfusion alterations in type 2 diabetes and its relation to insulin resistance and cognitive dysfunction

**DOI:** 10.1007/s11682-016-9583-9

**Published:** 2016-10-06

**Authors:** Ying Cui, Xia Liang, Hong Gu, Yuzheng Hu, Zhen Zhao, Xiang-Yu Yang, Cheng Qian, Yihong Yang, Gao-Jun Teng

**Affiliations:** 1 Jiangsu Key Laboratory of Molecular and Functional Imaging, Department of Radiology, Zhongda Hospital, Medical School of Southeast University, 87 Dingjiaqiao Road, Nanjing, Jiangsu 210009 China; 2 Neuroimaging Research Branch, Intramural Research Program National Institute on Drug Abuse, National Institutes of Health, 251 Bayview Blvd, Baltimore, MD 21224 USA

**Keywords:** Cerebral perfusion, Type 2 diabetes, Insulin resistance, Cognitive impairment, Arterial spin-labeling MRI

## Abstract

To explore the effect of T2DM on cerebral perfusion, and the relationship between cerebral perfusion changes and cognitive impairment as well as diabetic variables, by using a whole-brain arterial spin-labeling (ASL) MRI technique. This prospective study was approved by the local institutional review board and was performed between November 2012 and October 2013. All subjects provided informed consent. Forty T2DM patients and 41 age-, sex- and education-matched healthy controls were included. Cerebral blood flow (CBF) map was obtained by pulsed ASL perfusion imaging at 3 T MRI. Voxel-wise comparisons on CBF maps with and without partial volume effects (PVEs) correction were performed between groups. Associations between CBF and cognitive functioning, and between CBF and diabetic variables were investigated by using voxel-wise, whole-brain correlation analyses. In T2DM patients, PVEs uncorrected CBF was decreased in the posterior cingulate cortex (PCC), precuneus and bilateral occipital lobe, and increased in the anterior cingulate cortex (corrected *P* < .05). These changes were largely unchanged after PVEs correction. Correlation analyses revealed that in patients, hypoperfusion in PCC and precuneus regions were related to higher insulin resistance level and deficits in clock-drawing performance, while the occipital hypoperfusion was associated with worse visual-memory performance, regardless of PVEs correction. The cerebral hypoperfusion pattern in T2DM resembles the pattern observed in the early stage of dementia, and increased insulin resistance might be an important risk factor as well as treatment target for such CBF dysregulation.

**Electronic supplementary material:**

The online version of this article (doi:10.1007/s11682-016-9583-9) contains supplementary material, which is available to authorized users.

## Introduction

Type 2 diabetes mellitus (T2DM) is a prevalent metabolic disorder characterized by insulin resistance. A convergence of evidences has suggested T2DM to be associated with increased risk for cognitive impairment and dementia (Geijselaers et al. 2015). In the general population, cerebral blood flow (CBF) dysregulation is considered a risk factor for developing cognitive dysfunction (Ishiwata et al. 2006; Jagust et al. 1997). A growing number of studies on T2DM patients have demonstrated an association between hyperglycemia and cerebrovascular pathology (Almdal et al. 2004; Biessels et al. 2002), which might lead to the chronic and insidious ischemia of the brain (Biessels et al. 2006). Therefore, disturbances in cerebral perfusion are often hypothesized an important etiology of cognitive deficits in T2DM subjects.

However, the relation between cerebral perfusion, cognition and T2DM has not been well documented. Results from current few studies are inconsistent, some of which reported various degrees of impaired hemodynamics or vasoreactivity (Brundel et al. 2012; Novak et al. 2006; Last et al. 2007), while the others observed no difference comparing with healthy cohorts (Rusinek et al. 2015; Tiehuis et al. 2008; Dandona et al. 1978). This discrepancy might on one hand reflect the methodological differences in assessing CBF; more importantly, these studies primarily focused on the overall changes in global or cortical CBF, which may overlook the potential regional-specific effects caused by T2DM. Compared with global/cortical CBF, regional measurements are probably more advantageous to reveal localized alterations, which is often the case observed in earlier stage of cognitive decline (Johnson et al. 2005). Given the usually intact general cognitive functioning in T2DM patients (Ryan and Geckle 2000), a voxel-wise CBF measurement might be of higher sensitivity and specificity in tracking the early effects of diabetes on cerebral perfusion.

Arterial spin labeling (ASL) perfusion MRI is a noninvasive and sensitive imaging technique for CBF measurement (Williams et al. 1992). It labels the arterial blood water in the brain and uses it as an endogenous tracer, providing unique advantages over other imaging modalities that are often limited by exogenous contrast agents and imprecise prediction based on macrovascular flow. Compared with blood-oxygen-level dependent (BOLD) contrast MRI widely used in neural activity detection, ASL MRI reflects a more direct neurovascular coupling and permits quantitative measurement of CBF, which is less confounded by head motion artifacts (Detre and Wang 2002). With these advantages, ASL imaging has been used in studies of cerebrovascular and psychiatric disorders, yielding reliable and reproducible quantitative CBF measurement (Johnson et al. 2005; Le Heron et al. 2014).

The present study, therefore, used whole-brain ASL MRI technique to explore the cerebral perfusion changes in T2DM, and their relationship with cognitive impairment. We studied both partial volume effects (PVEs)-uncorrected and PVEs–corrected CBF maps to account for potential confounds by cortical atrophy. We hypothesized that 1) T2DM patients would exhibit alterations in cerebral perfusion, which is independent of cortical volume change; 2) the altered perfusion would correlate with impaired cognitive performance and specific diabetic variables in T2DM patients.

## Methods

### Subjects

This prospective study was approved by the local Research Ethics Committee and was performed between November 2012 and October 2013. All subjects provided their written informed consent before participation. Forty diabetic patients and 41 healthy controls were recruited from the local hospital and community via advertisement, respectively. Inclusion criteria included right-handedness, aged 50–70 years, and at least 6 years of education. Exclusion criteria consisted of alcohol or substance abuse, Mini-Mental State Examination (MMSE) score < 24, Hamilton Depression Rating Scale (HAM-D) score ≥ 7, past or current brain lesion, unrelated psychiatric or neurological disorder and MRI contraindications.

Diagnosis of T2DM was based on the latest criteria of American Diabetes Association (American Diabetes 2012). All patients were under close self-monitoring and received treatments including diet restriction, insulin, oral medications or combination therapy, with none having current or past history of hypoglycemia. To minimize the heterogeneity, patients who reported or were diagnosed with diabetic complications such as retinopathy, nephropathy, or neuropathy were excluded. Healthy subjects were matched with patients on age, gender and years of education.

### Biometric measurements

Blood samples were collected after overnight fasting to acquire FPG, HbA1c, fasting insulin and lipid level. Controls with fasting plasma glucose (FPG) level > 5.6 mmol/l or a 2 h postprandial glucose level > 7.8 mmol/l were considered at pre-diabetes state (American Diabetes 2012) and excluded from the study. During the visiting day, medical history, medication use, body weight, height, waist circumferences and blood pressure were also measured and recorded. Insulin resistance (IR) was determined by homeostasis model assessment of insulin resistance (HOMA-IR) for all participants except for those with insulin treatment.

Cognitive assessment covering several domains was performed by an experienced neurologist blind to the group allocation. Episodic memory regarding verbal and visual information was assessed by the Auditory Verbal Learning Test (AVLT) and Rey-Osterrieth Complex Figure Test (CFT)-delayed recall trial, respectively; working memory was measured by the forward and backward trials of the digit span test (DST); attention was evaluated by the Trail Making Test part A (TMT-A); executive functioning was assessed by Trail Making Test part B (TMT-B); visuo-spatial and attention was assessed by the Clock Drawing Test (CDT) and the CFT-copy trial.

### Image acquisition

MRI scanning was conducted on a Siemens 3 T Trio scanner (Erlangen, Germany). Subjects were instructed to keep their eyes closed, remain awake, avoid specific thoughts and keep their heads still during the scanning. ASL sequence was acquired by the following parameters: slice =27, repetition time (TR) = 4000 ms, echo time (TE) = 12 ms, slice thickness = 4 mm, flip angle =90°, field of view =220 mm × 220 mm, acquisition matrix =64 × 64, number of controls/labels =52 pairs. T1-weighted magnetization-prepared rapid gradient-echo imaging (MPRAGE) sequence was acquired to facilitate functional image preprocessing: section =176, TR = 1900 ms, TE = 2.48 ms, slice thickness = 1.0 mm, flip angle =9^o^, field of view =250 mm × 250 mm, acquisition matrix =256 × 256. Finally, fluid-attenuated inversion recovery (FLAIR) images were obtained: TR = 8500 ms, TE = 94 ms, slice =20, slice thickness = 5 mm. The potential small vessel disease (SVD) defined as white matter hyperintensity (WMH) and lacunar infarcts were evaluated on the FLAIR images as previously described (Wahlund et al. 2001). Briefly, the brain was divided into five regions on each hemisphere, and the WMH score was rated on each region separately on a 4-point scale (from 0 to 3), resulting in a final score ranged from 0 to 30. Participants with a score of 3 in any region were considered to have severe SVD and were thus excluded.

### Data processing

ASL data were analyzed using the Analysis of Functional Neuroimages (AFNI) software (National Institute of Mental Health, Bethesda, Maryland) (Cox 1996). At first, control and label ASL images were co-registered to each other, with head motion corrected and were smoothed with a 6-mm Gaussian kernel. The CBF-weighted time series were obtained by surround subtraction of the control and label images using sinc-interpolation. Quantitative CBF was calculated using the following model (Wang et al. 2003):

$$ {f}_{pASL}\left(x,y,z\right)=\frac{\lambda \cdot \varDelta M\left(x,y,z\right)}{2\alpha {M}_0\left(x,y,z\right)T{I}_1 \exp \left(-\frac{T{I}_2}{T_{1B}}\right)} $$

f_pASL_(x, y, z) is the blood flow at voxel (x,y,z) in milliliters per minute per 100 g brain tissue; ΔM is the CBF-weighted images and M_0_ is the fully relaxed image intensity; blood/tissue water partition coefficient λ = 0.9 mg/L; inversion efficiency α = 95 %; duration between inversion and saturation pulses TI_1_ = 600 ms; image acquisition time *TI*_2_ = 1600 ms; longitudinal relaxation time of blood T_1B_ = 1.624 s at 3.0 Tesla (Lu et al. 2004). Afterwards, the quantitative CBF image was co-registered to each subject’s T1-weighted image and spatially normalized to the Montreal Neurologic Institute (MNI) standard space with a resolution of 3 × 3 × 3 mm^3^.

To obtain perfusion information from primarily cortical regions, T1-weighted images were segmented to create probabilistic gray matter (GM) and white matter (WM) maps using Statistical Parametrical Mapping 8 (SPM8) software (http://www.fil.ion.ucl.ac.uk/spm/). The GM map was resampled to the resolution of the perfusion image and converted to a binary mask thresholded at 20 % probability to exclude voxels with small amounts of GM and then applied on the perfusion data. Finally, to reduce the effects of inter-subject variations in global CBF that is associated with non-neural factors and noise, the resultant CBF value was normalized by the global GM mean CBF, yielding an uncorrected relative CBF (CBF_uncorr_) map. This normalization strategy is widely applied in PET and ASL studies to improve sensitivity (Borghammer et al. 2008).

### PVEs correction

In CBF calculation, correction for partial volume effects (PVEs) is often performed to account for potential GM atrophy that may result in biased perfusion in voxels with greater volume fractions of WM and CSF (Johnson et al. 2005). We therefore performed the PVEs correction using the following formula (Johnson et al. 2005): SI_corr_ = SI_uncorr_ / (GM + 0.4 × WM), where SI_corr_ and SI_uncorr_ are corrected and uncorrected signal intensities. A similar normalization strategy was also applied as during the CBF_uncorr_ calculation, yielding the corrected relative CBF (CBF_corr_) map for each subject.

### Statistical analysis

#### Clinical characteristics

Statistical analyses were performed using SPSS software (ver. 18.0; SPSS, Inc., Chicago, IL, USA). Normal distribution was tested by the Kolmogorov-Smirnov test. Group comparison of clinical parameters were conducted using independent two-sample t-test for normally distributed variables, nonparametric Mann-Whitney U test for asymmetrically distributed variables, and χ2-test for categorical variables. A two-tailed *P* < 0.05 was considered statistically significant.

### Voxel-wise analysis of ASL data

Group average CBF_uncorr_ and CBF_corr_ value in each group were first calculated. Group effects on both CBF maps were analyzed using a voxel-wise general linear mixed-effects model, with age, gender and years of education as covariates. The education level was treated as a covariate of no interest to minimize its potential effects on cerebral perfusion as well as brain size (Chiu et al. 2004; Coffey et al. 1999). To exclude the confounding effects of SVD, presence of hypertension, hyperlipidemia, lacunar infarcts and WMH score were further controlled. The comparison was confined in GM mask obtained from the overlap of segmented GM images of all subjects. Statistical significance was set at two-tailed *P*_corrected_ < 0.05 (uncorrected *P* < 0.01 with a cluster of 53 voxels, determined by AFNI AlphaSim correction).

### Gray matter voxel-based morphometry analysis

Cortical volume changes were explored by voxel-based morphometry (VBM) analysis based on SPM8 software. The segmented GM maps in MNI space were modulated and smoothed with 8-mm Gaussian kernel. The maps were then compared between groups, with two-tailed *P* < 0.05 as statistical significance (uncorrected *P* < 0.01 combined with a cluster size of 232 voxels). Brain volumes in GM, WM and CSF as well as the total brain volume (sum of the three parts) were also compared.

### Correlations between perfusion and clinical data

The correlations between CBF maps (both uncorrected and corrected), diabetes variables (i.e., FPG, HbA1c and HOMA-IR) and neurocognitive performance that were significantly differed between groups (i.e., CFT-delay, CDT and TMT-B) were explored in a who-brain linear regression model, using the AFNI’s 3d LME command. The CBF maps and clinical variables in both groups were entered into the models. To identify the regions where the perfusion was differentially correlated with clinical variables in patients vs. controls, we focused on the group × variable statistical maps (two-tailed *P*_*corrected*_ < 0.05, *P*_*voxel-wise*_ < 0.01). Brain regions with significant interaction effects were considered important in contributing to the T2DM-related cognitive impairment. All analyses were adjusted for the same covariates as during the group comparison.

Additionally, to investigate the effects of glycemic control and treatment modality on brain perfusion, patients were either stratified according to HbA1c level (above and below 7 %) or treatment modality (diet restriction, oral hypogelycemic agents and insulin). The mean CBF_corr_ and CBF_uncorr_ values were extracted from each region with group differences and compared among these sub-groups.

## Results

### Demographic and cognitive characteristics

None of the participants was excluded because of severe SVD. The demographic and cognitive characteristics were summarized in Table 1. T2DM patients and controls were well matched for age, gender, education, head motion, blood lipid level, blood pressure, WMH and lacunar infarcts. As expected, the FPG, HbA1c and HOMA-IR level in the T2DM group were significantly higher (*P* < 0.05). Patients performed significantly worse on CFT-delay, TMT-B and CDT (*P* < 0.05), which involve multiple cognitive domains including memory, executive functioning and spatial processing. In T2DM group, disease duration was negatively correlated with performance in CFT (*R* = −0.496, *P* < 0.01) and CFT-delay (*R* = −0.35, *P* = 0.02). No correlations were found among other clinical parameters.

**Table 1:** Demographics and clinical characteristics for T2DM and control groups

Measures	T2DM (*n* = 40)	Control (*n* = 41)	*P* value
Age (years)	60.5 ± 6.9	57.9 ± 6.5	0.85
Gender (male/female)^a^	21/19	13/28	0.07
Education (years)	10.0 ± 3.4	10.3 ± 2.3	0.28
Head motion (FD value)	0.08 ± 0.04	0.07 ± 0.03	0.81
Diabetes duration (years)	8.9 ± 5.0	–	–
Insulin treatment (n)	8	–	–
HbA1c (%, mmol/mol)^b^	7.7 ± 1.6 (60.7 ± 16.4)	5.6 ± 0.3(37.7 ± 3.3)	<0.01
FPG (mmol/L)^b^	7.8 ± 2.1	5.4 ± 0.3	<0.01
HOMA-IR^b^	3.3 ± 1.9	2.4 ± 1.1	0.02
BMI (kg/m^2^)	24.4 ± 2.7	23.8 ± 2.6	0.29
Systolic BP (mmHg)	136.6 ± 14.6	132.5 ± 15.0	0.21
Diastolic BP (mmHg)	86.0 ± 10.9	86.9 ± 11.3	0.77
Total cholesterol (mmol/L)	5.5 ± 1.1	5.3 ± 0.8	0.46
Triglyceride (mmol/L)	1.5 ± 0.8	1.3 ± 0.7	0.30
White matter lesions (range)	0–6	0–7	0.07
Lacunar infarcts (n)^a^	9	5	0.25
Cognitive performance			
MMSE	28.3 ± 1.0	28.6 ± 1.2	0.09
AVLT	5.9 ± 1.4	6.5 ± 2.1	0.16
AVLT-delay	5.8 ± 2.4	6.3 ± 2.1	0.27
CFT-delay^b^	13.7 ± 5.5	17.3 ± 5.9	0.01
DST (forward)	6.9 ± 1.2	7.3 ± 1.6	0.37
DST (backward)	4.2 ± 0.9	4.5 ± 1.3	0.25
TMT-part A (s)	63.7 ± 12.7	63.3 ± 15.1	0.90
TMT-part B (s)^b^	174.3 ± 53.3	154.5 ± 50.1	0.02
CFT-copy	34.5 ± 1.5	35.0 ± 1.5	0.24
CDT^b^	3.2 ± 0.6	3.5 ± 0.6	0.04
VFT	16.5 ± 3.6	17.1 ± 3.2	0.48

Data are represented as mean ± (SD), n or range. ^a^ The statistical analyses were performed by χ^2^ test. ^b^ *P* value <0.05. FPG, fasting plasma glucose; HOMA-IR, homeostasis model assessment of insulin resistance; BMI, body mass index; BP, blood pressure; MMSE, mini-mental state examination; AVLT, auditory verbal learning test; CFT, complex figure test; DST, digit span test; TMT, trail-making test; CDT, clock drawing test; VFT, verbal fluency test

### Voxel-wise CBF differences

**Fig. 1:**
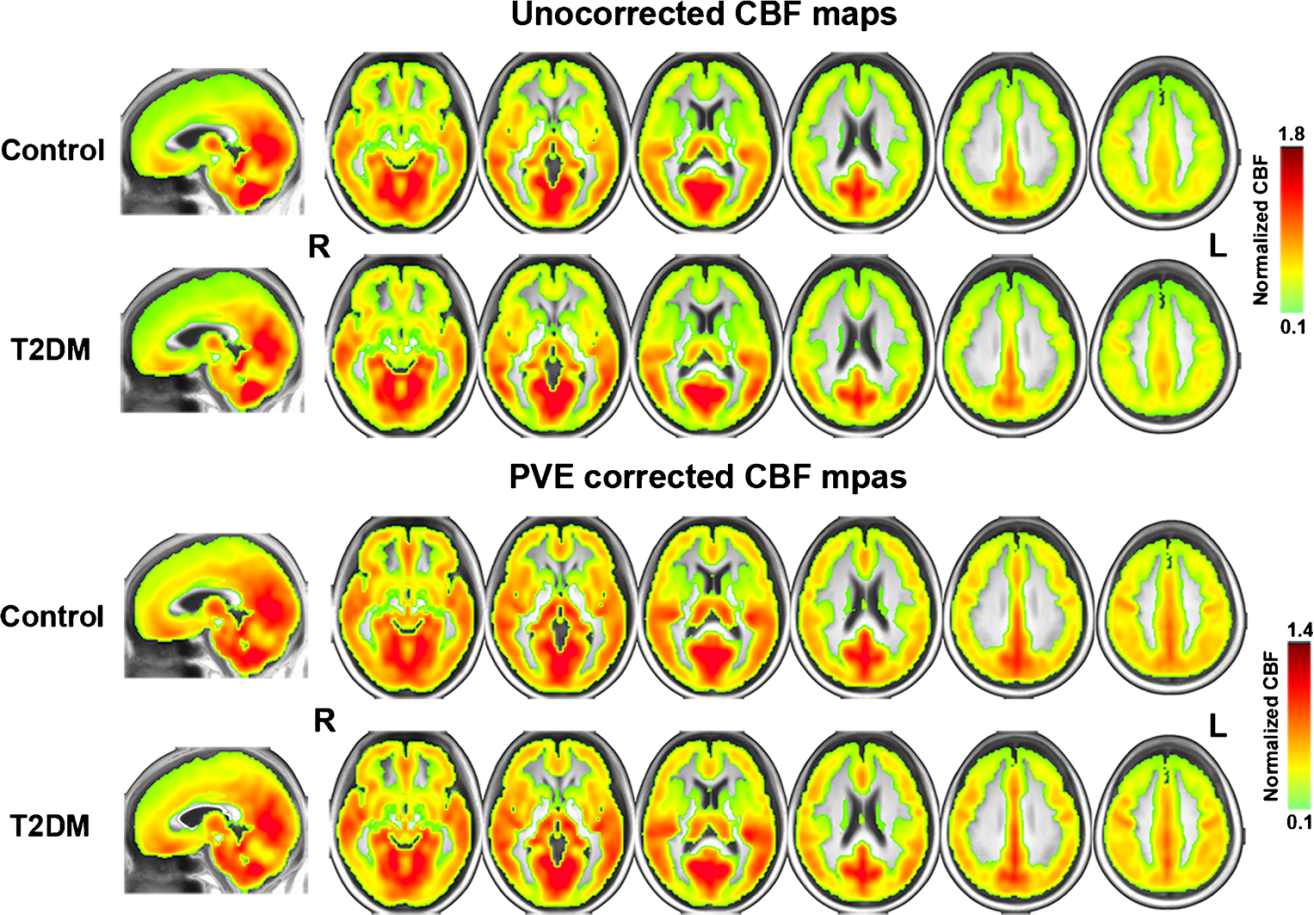
Group average CBF_corr_ and CBF_uncorr_ maps in T2DM and control group. Within each group, the average relative CBF_uncorr_ and CBF_corr_ values in PCC, precuneus, adjacent visual cortex, MPFC and temporal regions are higher than other brain regions. Note that the perfusion in T2DM group, especially in the precuneus and occipital regions, is lower than the control group (arrows). Color scale denotes the relative CBF value after normalization with the global mean CBF. The maps are in Montreal Neurologic Institute standard space. R, right; L, left. PCC, posterior cingulate cortex; MPFC, medial prefrontal cortex

The average CBF_uncorr_ and CBF_corr_ maps are shown in Fig.1. In each group, perfusion in occipital lobe, temporal lobe and the putative default-mode network (DMN) regions including posterior cingulate cortex (PCC), precuneus and MPFC were higher than other regions, which is consistent with previous results (Liang et al. 2013).

**Fig. 2:**
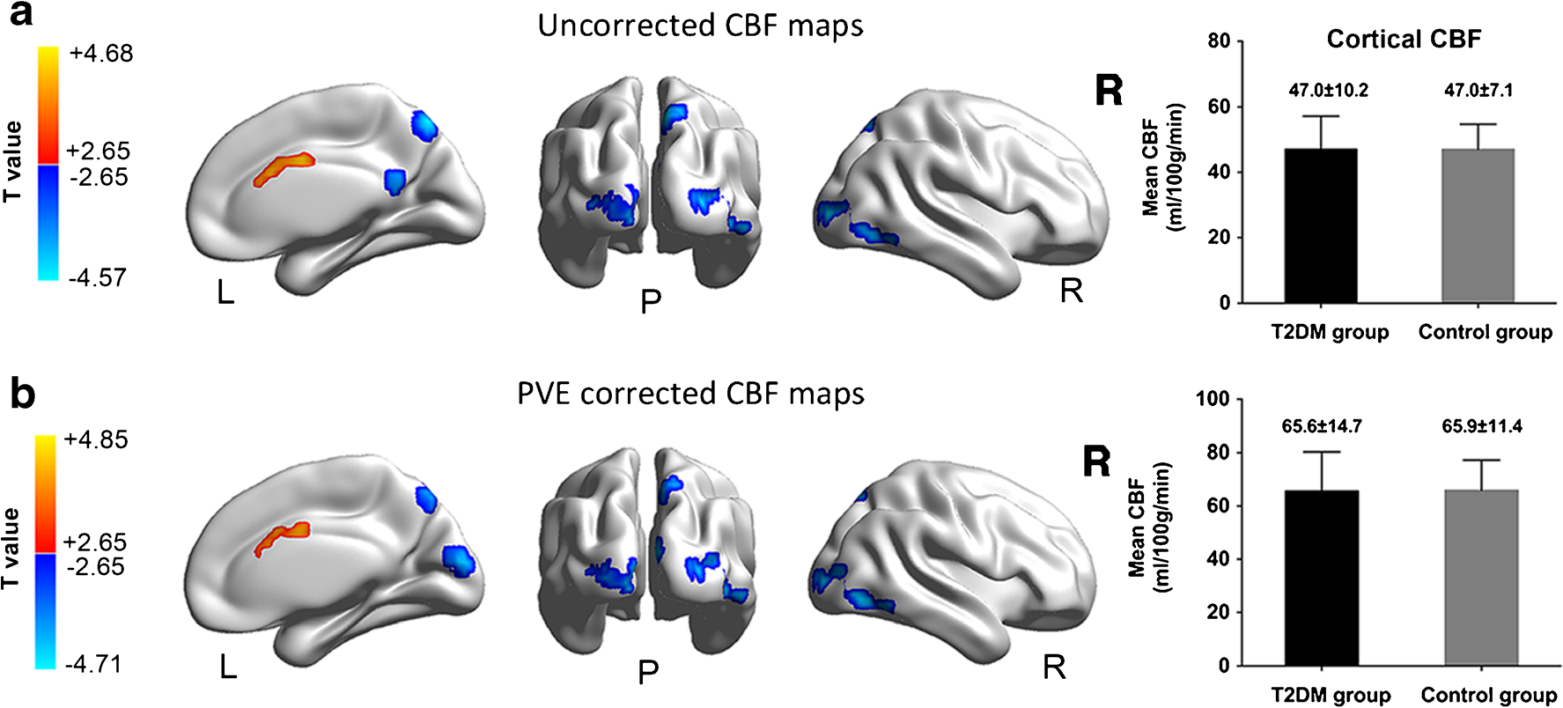
Group differences of CBF_uncorr_ and CBF_corr_ maps between T2DM patients and healthy controls (*P* < 0.05, Alphasim corrected). Compared to healthy controls, T2DM patients showed significantly lower CBF_uncorr_ in the right PCC, precuneus and bilateral MOG (Fig. 2a, cool color). On the other hand, patients showed higher CBF_uncorr_ in the dACC (Fig. 2a, warm color). The results in precuneus and MOG were largely remained after accounting for the PVEs (Fig. 2b), but the PCC did not survive after the multiple comparison correction. The results were mapped on cortical surfaces using the BrainNet viewer (www.nitrc.org/projects/bnv). Color scale denotes the *T* value. R, right; L, left; P, posterior. PCC, posterior cingulate cortex; MOG, middle occipital gyrus; dACC, dorsal anterior cingulate cortex

Compared with controls, T2DM patients showed decreased CBF_uncorr_ in PCC, precuneus and bilateral middle occipital gyrus (MOG) (Fig. 2a, cool color), but increased CBF_uncorr_ in the dorsal anterior cingulate cortex (dACC) (Fig. 2a, warm color). These results remained largely unchanged after the PVEs correction, except that the cluster size of PCC shrunk and did not survive the multiple comparison correction (Fig. 2b). A detailed list of the identified brain regions is summarized in Table 2. Notably, the global average CBF did not show significant group difference, indicating the early effects of T2DM on brain perfusion being more localized rather than globalized (Fig. 2, right column).

**Table 2:** Brain regions with significant differences in the CBF_uncorr_ and CBF_corr_ maps between T2DM patients and healthy controls (corrected *P* < 0.05)

Brain regions	MNI			Voxels	Peak *T* value
	x	y	z		
Uncorrected CBF map					
Dorsal ACC	+3	0	+33	124	+4.68
R MOG	+24	-93	+3	161	-4.87
L MOG	-21	-96	+3	139	-4.21
R Precuneus	+9	-69	+51	77	-4.35
R PCC	+9	-51	+21	53	-4.18
PVE corrected CBF map					
Dorsal ACC	+9	+9	+30	90	+5.00
R MOG	+24	-93	+3	155	-4.88
L MOG	-21	-93	+3	123	-4.24
R Precuneus	+9	-69	+51	52	-4.10
Cuneus	+9	-84	+15	60	-4.82

Comparisons were performed at *P* < 0.05, corrected by family-wise multiple comparison correction (AFNI software). MNI, Montreal Neurological Institute; x, y, z, coordinates of primary peak locations in the MNI space; positive *T* values: T2DM > control subjects; negative *T* values: T2DM < control subjects. ACC, anterior cingulate cortex; PCC, posterior cingulate cortex; MOG, middle occipital gyrus; R, right; L, left

### Gray matter voxel-based morphometry analysis

During the VBM analysis, no suprathreshold voxel-wise difference was observed in the GM concentration between the two groups. Through brain volume comparison, we found that T2DM patients showed a larger CSF volume than the HCs. However, the GM, WM and total volume did not differ between groups (Supplementary Table 1).

### Associations between CBF and clinical measurements

**Fig. 3:**
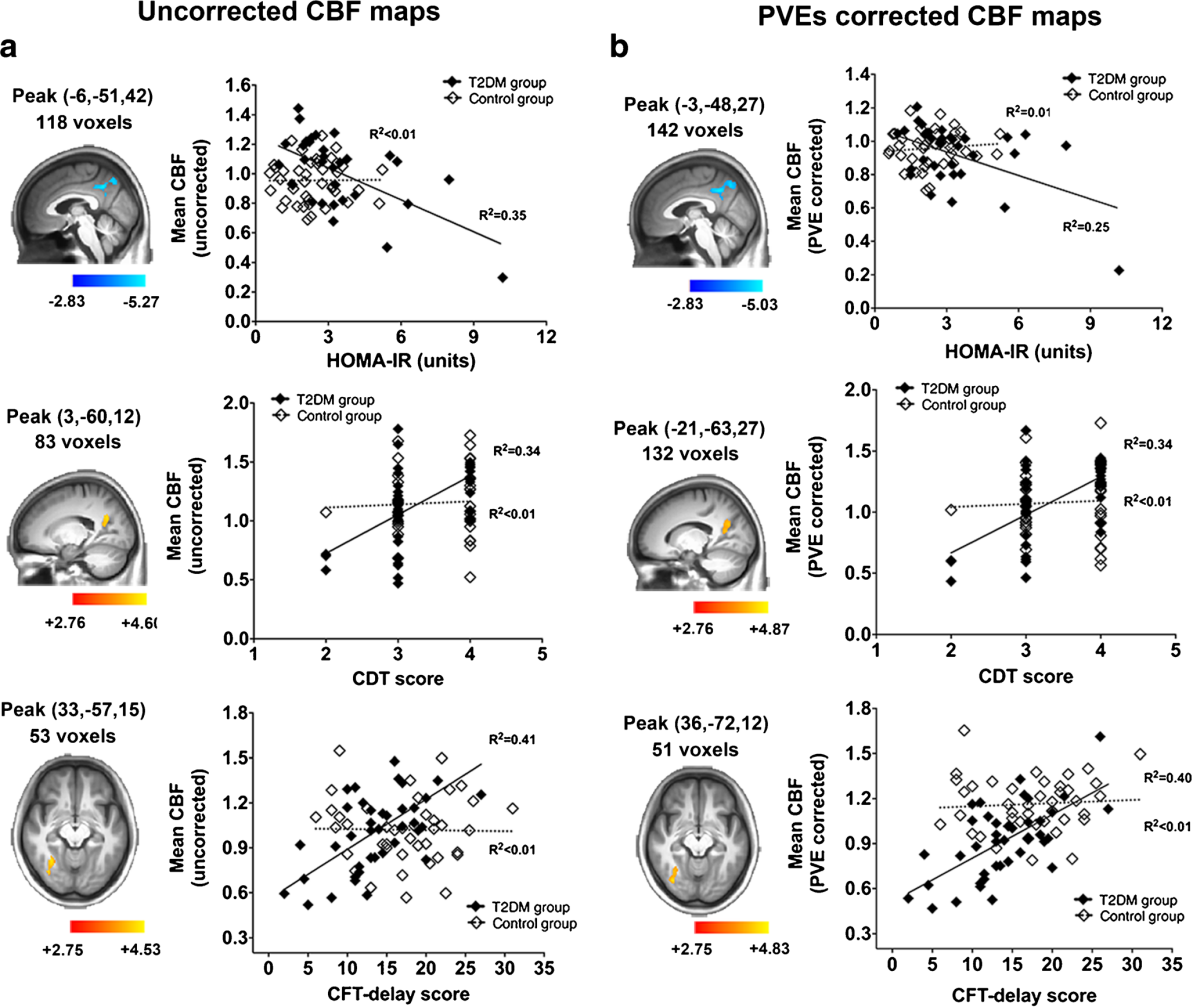
Voxel-wise correlations between CBF maps and clinical parameters. Results of the CBF_uncorr_ map are shown in the Fig. 3a, while the results of CBF_corr_ map are shown in Fig. 3b. HOMA-IR index was negatively correlated with the perfusion in posterior regions, especially PCC and precuneus (R^2^ = 0.35 [CBF_uncorr_ map], R^2^ = 0.25 [CBF_corr_ map]) (first row); CDT score was positively correlated with the perfusion in PCC/precuneus region (R^2^ = 0.34 [CBF_uncorr_ map], R^2^ = 0.34 [CBF_corr_ map]) (second row). CFT score was positively correlated with the perfusion in the right middle/inferior occipital gyrus (R^2^ = 0.41 [CBF_uncorr_ map], R^2^ = 0.40 [CBF_corr_ map]) (third row). HOMA-IR, homeostasis model assessment of insulin resistance; PCC, posterior cingulate cortex; dACC, dorsal anterior cingulate cortex. White squares and dotted line, Control group; Black squares and solid lines, T2DM group

During the correlational analyses, no group × variable interaction effect was detected in any of the clinical variables. We next performed the voxel-wise correlational analyses using the same clinical variables and covariates as in the interaction model in each group separately. In T2DM group, disease duration was also included in the clinical variables. Due to the significant relationship between disease duration and CFT-delay performance, the former was further controlled in addition to the original covariates while exploring the correlation between CBF and the related performance. In T2DM group, HOMA-IR was negatively correlated with the perfusion in extensive posterior regions, especially in the precuneus and PCC (R^2^ = 0.35 [CBF_uncorr_ map], R^2^ = 0.25 [CBF_corr_ map]) (Fig.3, first row). Performance in CDT was also positively correlated with the perfusion in similar regions, especially the PCC region (R^2^ = 0.34 for both maps) (Fig.3, second row). Besides, the CFT-delay performance was correlated with the occipital perfusion after additionally controlling for the disease duration (R^2^ = 0.40 [CBF_uncorr_ map], R^2^ = 0.41 [CBF_corr_ map]) (Fig.3, third row). Notably, no such voxel-wise correlation was observed between CBF with other clinical variables nor in the control group.

Finally, patients were stratified according to either the HbA1c level or treatment modality to examine the contribution of these clinical parameters to the perfusion alterations. The sample size for the patients having an HbA1c less than 7 % was 17, while the number of those having an HbA1C of above 7 % was 23. For the sub-group treated with insulin, the sample size was 8. The remainders were either treated with oral hypoglycemic agents (*n* = 22) or diet restriction only (*n* = 10). Results suggested a trend towards lower PCC/cuneus, precuneus and occipital perfusion in patients with poorer glycemic control (HbA1c > 7 %) (Supplementary Fig. 1, a and c). A similar trend was also observed among groups with different treatment modalities, where insulin-treated patients showed the lowest perfusion in bilateral MOG and dACC when comparing with those treated with oral hypoglycemic agents and diet restriction (Supplementary Fig. 1, b and d). However, none of these results reached statistical significance.

## Discussion

The major findings of the present study are as follows: first, by using ASL perfusion MRI, we identified brain regions with hypoperfusion in T2DM patients, including the PCC, precuneus, and bilateral MOG, which are important structures that often show vulnerability in the early process of neurodegeneration. Second, the observed perfusion changes were independent of potential cortical atrophy, as no cortical volume difference was detected and the hypoperfusion pattern largely remained after PVE correction. Finally, strong correlations were observed between posterior hypoperfusion and IR, and between the hypoperfusion and impaired cognitive performance in T2DM patients. Taken together, our findings of cerebral hypoperfusion might provide valuable insights into the neural substrate of T2DM-associated cognitive impairment.

The PCC and precuneus are core regions of the well-defined default mode network (DMN), whose dysfunctions are considered hallmarks of neurodegenerative diseases (Greicius et al. 2004). ASL studies on AD patients showed that cerebral perfusion reductions were most prominent in the parietal cortices and PCC area, especially in patients at an earlier stage (Johnson et al. 2005). Other neurodegenerative conditions such as aging and Parkinson’s disease also showed similar posterior hypoperfusion patterns (Liu et al. 2012; Le Heron et al. 2014). Similarly, parallel regions are also reported abnormal in T2DM patients: previous study on a similar patient group found decreased neural intensity and coherence around the precuneus regions (Cui et al. 2014); a recent PET study involving early T2DM patients reported hypometabolism in preceneus and PCC regions that were related to higher insulin resistance level (Baker et al. 2011). Given its consistency across neurodegenerative conditions, such posterior hypoperfusion holds the potential to serve as early biomarker of the cognitive impairment in T2DM. However, it is noteworthy that the PCC did not survive after the PVE correction, suggesting that PCC might undergo a milder perfusion change than other posterior regions. Further studies are warranted to study the progression of hypoperfusion in more advanced patients.

Noteworthy is that the hypoperfusion in precueneus and PCC was significantly correlated with higher HOMA-IR level. Insulin modulates various physiological activities in the brain and is supportive of normal cognitive functioning such as learning and memory, the reduction of which may interfere with the glucose use and lead to neuronal dysfunction (Lucignani et al. 1987). Therefore, IR has been increasingly recognized to be an independent risk factor for developing cognitive impairment (Biessels and Reagan 2015). As both the causal factor and central characteristics of T2DM, IR is therefore consistently suggested as an important etiology for the onset and development of cognitive impairment (Duarte 2015). In our results, IR in T2DM group is exclusively correlated with the posterior regions that are most vulnerable in MCI and subjects at risk for AD, suggesting that IR might be the key link for the increased incidence of AD in individuals with T2DM (Kim and Feldman 2015).

Significant occipital hypoperfusion was also observed. In T2DM patients, occipital areas have shown not only reduced cerebrovascular reactivity, but also decreased neural activity and brain volumes (Tchistiakova et al. 2014; Cui et al. 2014; Espeland et al. 2013). These abnormalities in occipital regions might be attributed to the potential visual impairment, a well-known complication of diabetes, which often involves vasculopathy and neuropathy along the visual pathway (Heravian et al. 2012). However, since none of our included patients manifested clinically measurable retinopathy or visual impairment, whether this occipital hypoperfusion results from visual impairment or a preferential posterior circulation impairment needs to be further examined.

On the other hand, increased rCBF was observed along the dorsal part of ACC, which is a critical region responsible for higher-order cognitive control (Sheth et al. 2012). Its hyperperfusion is often observed in mild cognitive impairment, assuming to represent a compensatory response to an encroaching neuropathology (Wierenga et al. 2012). Given that the cognitive deficits are more prominent in elder T2DM patients (>65 years old) than those at younger age (Ryan and Geckle 2000), the elevated dACC perfusion in the present study might reflect a vascular response to the increased need of glucose and oxygen to achieve a normal cognitive performance during the early stage. As lower dACC perfusion was observed in more advanced patients (Supplementary Fig. 1b), longitudinal studies are promising to determine whether there is a breakdown in this compensatory mechanism in progressive patients.

In the current results, the hypopefusion pattern in T2DM was largely remained after the PVEs correction, which might be attributed to the insignificant difference in the voxel-based morphometry in GM as well as the total GM volume between the two groups. More importantly, the remained results also suggested that the hypoperfusion in T2DM patients were not caused by the PVEs in the setting of potential cortical atrophy. This is consistent with previous studies with subjects at high risk for developing dementia, suggesting that perfusion deficits may exist in presymptomatic stages before substantial atrophy is present (Johnson et al. 2005; Xekardaki et al. 2015). However, noteworthy is that the formula we used for the PVEs correction was based on the spatial resolution that is different from ours (Johnson et al. 2005), which might potentially confound the estimation of the corrected CBF values. The more rigorous algorithm proposed by Asllani et al. (Asllani et al. 2008) that reflects the pure tissue contribution instead of the GM-to-WM flow ratios should be considered as an alternative approach to overcome such limitation.

There are limitations to this study. First, the relatively small sample size limited our ability to identify the CBF differences among sub-groups of patients and to examine the relationship between CBF and other clinical variables. More patients should be included in future studies. Second, additional hemodynamic measurements such as vascular reactivity were not assessed. Inclusion of such measurements could further facilitate our understanding of the vascular pathology in T2DM patients. Finally, the statistical power of the current study could be reduced by the heterogeneous medication of the included participants. Further studies with non-treated subjects are warranted to exclude such confounding effects.

In conclusion, the present study examined the regional effects of T2DM on cerebral perfusion. Hypoperfusion was observed primarily in the PCC, precuneus and bilateral occipital lobe, which was largely independent of potential cortical atrophy. Importantly, the hypoperfusion in the PCC and precuneus was strongly correlated with increased IR level and cognitive deficits, suggesting such hypoperfusion pattern as a promising biomarker for cognitive impairment in T2DM patients. Future studies are merited to explore the role of IR-targeted treatment in the modulation of cerebral CBF and prevention of dementia development in diabetic population.

## Electronic supplementary material

ESM 1 (DOCX 10502 kb)
