# Supplementary material for: Cerebral perfusion alterations in type 2 diabetes and its relation to insulin resistance and cognitive dysfunction
Source: Brain Imaging Behav. 2016 Oct 6;11(5):1248–57. doi: 10.1007/s11682-016-9583-9 (PMC5653700; doi:10.1007/s11682-016-9583-9)
Supplement: Supplementary file 1 — (DOCX 10502 kb) [file 11682_2016_9583_MOESM1_ESM.docx]

Cerebral perfusion in type 2 diabetes and its relation to insulin resistance and cognitive dysfunction

Journal name: Brain imaging and behavior

Ying Cui, MD ^1,2^; Xia Liang, PhD ^2^; Hong Gu, PhD ^2^; Yuzheng Hu, PhD ^2^; Miao Yu, MD ^1^; Zhen Zhao, MD ^1^; Xiang-Yu Yang, MD^1^; Cheng Qian, MD^1^; Yihong Yang, PhD ^2^; Gao-Jun Teng, MD *^1^

^1^ Jiangsu Key Laboratory of Molecular and Functional Imaging, Department of Radiology, Zhongda Hospital, Medical School of Southeast University, Nanjing, China

2 Neuroimaging Research Branch, National Institute on Drug Abuse, National Institutes of Health, Baltimore, MD, USA

**Corresponding author:* Gao-Jun Teng

Jiangsu Key Laboratory of Molecular and Functional Imaging, Department of Radiology, Zhongda Hospital, Medical School of Southeast University, Nanjing, China

E-mail: [gjteng@vip.sina.com](mailto:gjteng@vip.sina.com)

**Supplementary Table 1.** **Group differences of brain volume between T2DM patients and healthy controls**

| **Brain volume** | **T2DM patients**  **(n=40)** | **Healthy control**  **(n=41)** | ***P* value** |
| --- | --- | --- | --- |
| **GM** | 728.1±38.8 | 741.8±35.2 | 0.10 |
| **WM** | 665.5±29.9 | 675.9±31.4 | 0.13 |
| **CSF** | 306.9±35.4 | 281.2±33.9 | 0.001* |
| **Total** | 1700.5±43.1 | 1698.9±35.4 | 0.86 |

* *P* < 0.05. GM, gray matter; WM, white matter; CSF, cerebrospinal fluid.

**Supplementary Fig. 1 Distribution of the brain volume in the T2DM and control groups**

**a, b and d**, the volume in gray matter, white matter and the total volume did not differ between the groups. **c**, the CSF volume was significantly higher in the T2DM group. No outliers were found in the distribution of the four variables in both groups.

**
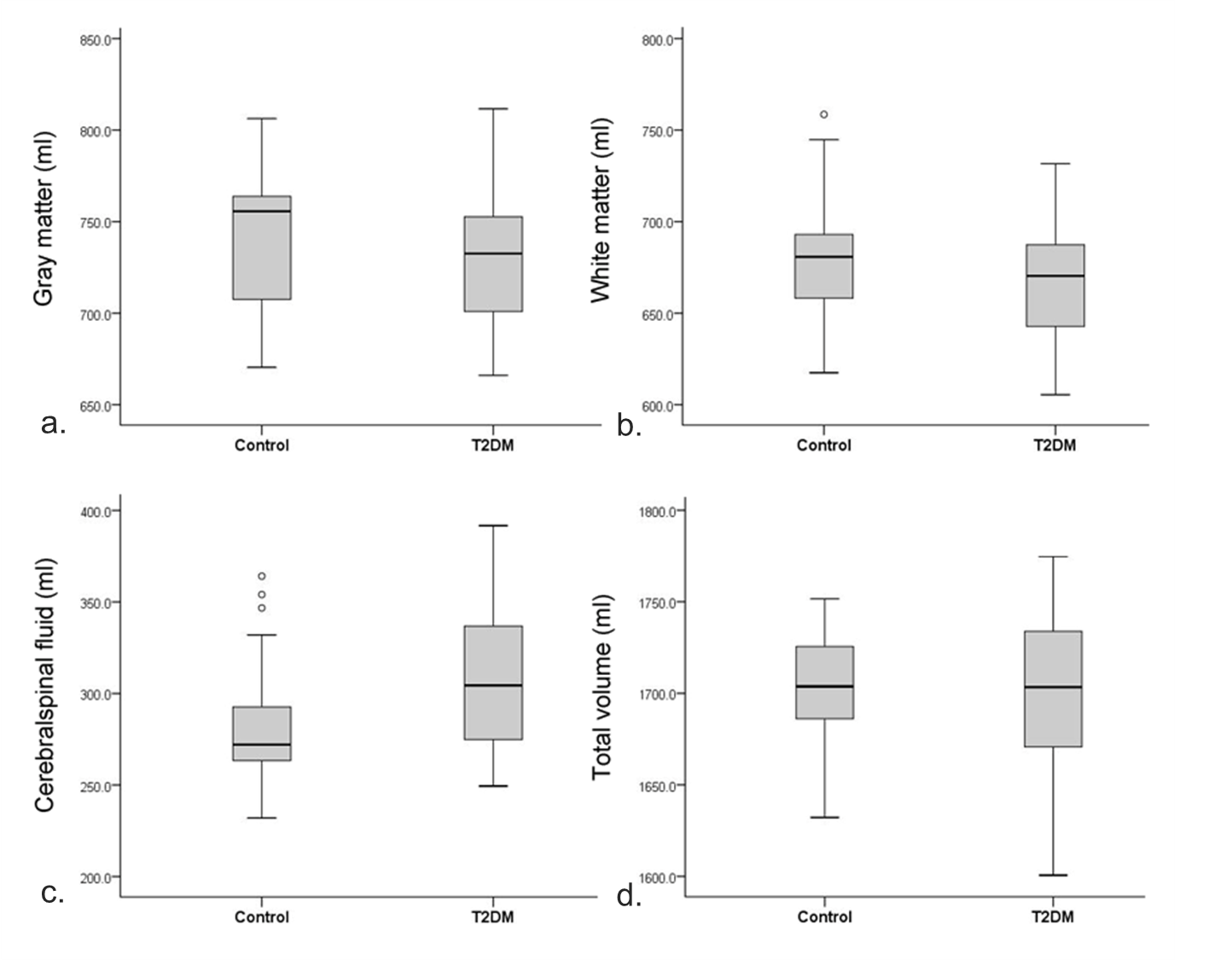
**

**Supplementary Fig. 2 Group differences of CBF among different sub-groups of T2DM patients.**

**a and c,** Although the perfusion difference did not reach statistical significance, there is a trend towards lower posterior perfusion in patients with poorer glycemic control (HbA1c > 7%) (black bar), in both CBF_uncorr_ and CBF_corr_ values. **b and d**, the mean CBF_uncorr_ and CBF_corr_ value also showed no difference among patients with different treatment modalities. However, there is a trend towards lower perfusion in dACC and posterior regions in patients with insulin treatment (black bar) comparing to those with no-treatment or oral hypoglycemics. Data are presented as mean ± (SE). dACC, dorsal anterior cingulate cortex; MOG, middle occipital gyrus; PCC, posterior cingulate cortex

**
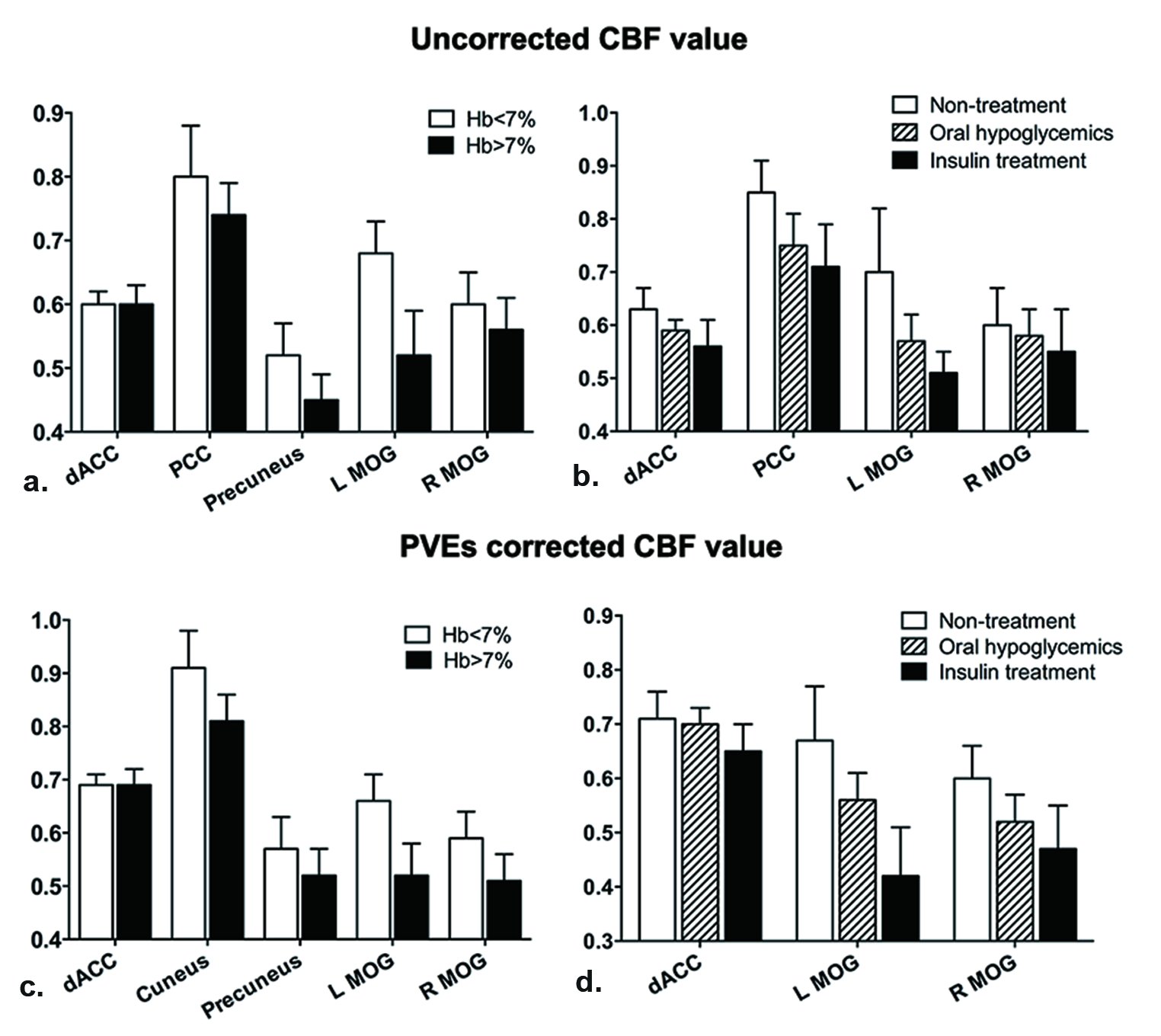
**
